# The impact of the COVID-19 pandemic on the mental health of children with psychiatric diagnoses – multidimensional CCPCA Model

**DOI:** 10.1186/s12888-022-04144-2

**Published:** 2022-07-23

**Authors:** Anna Maria Kalenik, Mariusz Topolski, Justyna Górnik, Tomasz Wolańczyk

**Affiliations:** 1 Department of Child Psychiatry, Medical University of Warsaw, Żwirki i Wigury 63A, 02-191 Warsaw, Poland; 2 Faculty of Information and Communication Technology, Wrocław University of Science and Technology, Wybrzeże Wyspiańskiego 27, 50-370 Wroclaw, Poland

**Keywords:** COVID-19, Children and adolescents, Psychiatric disorder, Class-Centroid Principal Component Analysis, CCPCA

## Abstract

**Background:**

The study aimed to assess the severity of symptoms of anxiety and depression in children with previously diagnosed psychiatric disorders during the COVID-19 pandemic in Poland.

**Methods:**

Online questionnaires were used to investigate three groups of subjects: patients with a psychiatric diagnosis, primary school pupils, and children from children’s homes. A total of 167 children with their parents or guardians participated in the study. In addition to basic statistics, a multidimensional Centroid Class Principal Component Analysis (CCPCA) model was used.

**Results:**

It was found that the strongest fear of the coronavirus was experienced by children from children’s homes, while the most severe depressive symptoms and state anxiety were observed among patients diagnosed with psychiatric disorders. Parental care by assisting with school education and lack of close contact with other people (less than two metres) at parents/guardians’ work had the most potent protective effect in reducing the fear of COVID-19.

**Conclusions:**

There is a need for further research in children and adolescents to develop effective strategies for protecting their mental well-being when faced with social isolation or disease.

## Background

The SARS-CoV-2 virus has been disturbing the world since 2019. The COVID-19 pandemic has affected not only the physical health of people but also the economy. Furthermore, the number of studies to suggest that it has had a significant impact on mental health is ever-increasing, irrespective of age, country, education background or wealth [1, 2]. This is not entirely surprising considering what we learned during previous epidemics and pandemics, including the SARS epidemic, the H1N1 influenza ('swine flu') pandemic or the Ebola epidemic [3–5]. At 1 month after hospitalisation for SARS, 10–18% of survivors reported symptoms of posttraumatic stress, anxiety and depression [3]. At the peak of the H1N1 pandemic, anxiety was common among college students [6]. Fear of the swine flu was found to be higher among students with obsessive–compulsive symptoms [7]. During the Ebola epidemic, fear of this disease was correlated with general distress, contamination cognitions and disgust sensitivity [8]. In studies among children, the fear of the swine flu was significantly related to their parents’ fear of the disease [9]. As reported by Sprang [10], criteria for post-traumatic stress disorder (PTSD) were met in as many as a quarter of isolated or quarantined children. In recent years there have been a number of reports on the deterioration of mental well-being in adults during the COVID-19 pandemic [1, 2, 11–13]. Some studies, though not many, have sought to explore also the paediatric population [2, 12, 14–16]. In a study of school students in Wuhan, an astounding 26.5% of the participants reported depressive symptoms and 19.6% reported anxiety symptoms [16]. The available literature puts forward that the well-being of children and adolescents may be affected by a number of factors during the pandemic. One of them is fear of the disease per se [17]. Fear for relatives and of their getting infected is apparently another major factor. One of the studies reported that children were not only afraid of infecting their grandparents, but would even have felt guilty had someone close to them become infected [18]. Younger children (3–6 years) were more likely to manifest fear that their family members could contract the infection [19]. Children may also be less informed than adults about the real risk. Younger children are particularly dependent on the information provided by their parents. Furthermore, the information they receive is both subjective and distorted by the anxiety and fear experienced by their parents or guardians [9, 20]. A study by Muris [21] concluded that children’s fear beliefs were determined by the negative narratives provided by their parents. As school consumes a substantial amount of time, school closures triggered a major shift in the life of children and adolescents [22]. Reorganisation of daily routines coupled with increased levels of stress and longer exposure to alerting ‘blue light’ (computer and tablet screens) due to remote learning may significantly alter circadian rhythms and cause or worsen sleep problems [23]. A study by Zreik [12] demonstrated that about 30% of mothers reported a negative change in child’s sleep quality and a decrease in sleep duration during the COVID-19 pandemic. Altered sleep patterns were even observed in preschoolers [24]. Sleep disorders are firmly associated with diminished mental comfort, poorer behavioural functioning, aggressive and risky behaviour, and attention deficit [25]. This seems crucial in view of the knowledge that sleep disturbance may be a predictor of subsequent depression [26] or may constitute a risk for other psychopathologies in children and adolescents [27]. Furthermore, it was observed during the COVID-19 outbreak that stress mediated the association between internet gaming disorder and both insomnia and quality of life [15]. Social isolation involves the absence of contact with peers, friends and colleagues, which is particularly relevant in adolescence [28, 29]. Simultaneously, the number of child helpline contacts related to violence increased in some countries [30]. A number of studies reported the negative impact of imposed quarantine, with some researchers suggesting its long-lasting effects [31].

Psychiatric disorders are generally known to predispose to higher vulnerability to the negative effects of external influences. In the pandemic conditions, patients may be more susceptible to stress, leading to relapses or worsening of pre-existing mental health condition [7, 32]. In Germany, adult patients suffering from mental illnesses scored significantly higher for COVID-19-related fear versus healthy individuals [33]. Mothers of autistic children, compared to mothers of healthy children, tended to have a higher level of health anxiety and a lower level of psychological well-being [34].

In addition to anxiety-depressive symptoms, it is also worth looking at obsessive–compulsive symptoms. These disorders are characterized by a significant comorbidity [35, 36]. Sources say that stress, trauma and difficult experiences may be a risk factor for the development of OCD, the appearance of additional symptoms or their severity [36–40]. During the COVID-19 pandemic, a positive correlation of symptoms of anxiety and depression with symptoms of OCD was noticed [38, 41, 42]. According to Turkish researchers, fear of COVID-19 in adolescents is a significant positive predictor of anxiety-depressive symptoms, which in turn also has a positive effect on OCD symptoms [38]. In addition, during the pandemic, for epidemiological reasons, frequent washing of hands, wearing of masks and social distancing were intensively promoted as a means of protection against infection. Many people experienced fear of falling ill and of losing loved ones followed by hygiene or collected food and medication [38, 43]. Such thoughts and behaviors related to excessive cleaning symptoms, fear of harm to oneself and others, and hoarding may also be an element of OCD [36]. Intensification of media and social pressure on intensive hygiene, as well as misinformation, may contribute to the appearance or worsening of symptoms of this disorder [38, 39, 43–45]. During the pandemic, worsening of OCD symptoms was observed in the Danish population of children and adolescents who were newly diagnosed or diagnosed many years before the pandemic [41]. Another study in the Turkish population in this age group also found that contamination obsessions and cleaning compulsions have intensified significantly [40]. However, the results were not homogeneous as a study of Israeli children and adolescents diagnosed with OCD did not show any worsening of symptoms, and even improved quality of life was observed in these patients [46].

However, in the available literature, relatively little attention has been paid to the mental condition of children with a psychiatric diagnosis during the COVID-19 pandemic [38, 40, 47]. As of the time of writing of this article, no studies pertaining to this population in Poland have been published. To address this gap, in our study we focused specifically on younger patients with a psychiatric diagnosis.

This study aimed to investigate selected components of children’s mental health during the SARS-CoV-2 pandemic. In addition to fear of the virus per se, we explored the severity of depressive and anxiety symptoms, and assessed the severity of obsessive–compulsive symptoms. We also attempted to establish which factors contributed significantly to these characteristics in the paediatric population.

## Methods

### Procedure

This was a non-interventional, survey-based, matched-cohort study. Ethical approval was exempted for the study by the Bioethics Committee of the Medical University of Warsaw. The participants completed online questionnaires with their parents or guardians during school closures in Poland, prompted by the COVID-19 pandemic. Responses were collected between 6 May and 25 June 2020. The first part of the questionnaire was addressed to the parents or guardians and included questions about the child’s sociodemographic characteristics (age, gender, residence), epidemiological situation (quarantine, infections in the family, social distancing, etc.), professional status (parents/guardians’ employment status), schooling situation (models of learning during the pandemic, helping with schoolwork), and psychological/psychiatric care provided to the child (before or during the pandemic). The second part was addressed to the children and incorporated the following diagnostic tools: the Fear of COVID-19 Scale (FCV-19S) [48, 49], the Children's Depression Inventory 2 (CDI-2) [50, 51], the State-Trait Anxiety Inventory for Children (STAIC) [52, 53], and the Leyton Obsessional Inventory-Child Version (LOI-CV) [54–56].

### Participants

The study group included patients treated in the inpatient and day care wards of the Child Psychiatry Department in Warsaw, hospitalised in 2019 or 2020 and aged 10–16 years (318 patients). The exclusion criteria were a diagnosis of intellectual disability or lack of fluency in Polish (34 patients). A total of 218 parents/guardians agreed to participate in the study.

The concurrent control group consisted of volunteers: sixth, seventh and eighth grade students of primary schools with their parents/guardians who agreed to participate in the study. The questionnaire was sent to pupils in six schools in the Mazovian Region of Poland (1179 students).

There was also a third cohort in the study, which consisted of children from children’s homes in Warsaw (born between 2003 and 2010) and their guardians.

Ultimately, we received 75 questionnaires completed by patients, 61 from the pupil group and 31 from children’s homes.

### Diagnostic tools used in the study

#### Fear of COVID-19 scale [48, 49]

The Polish version of the Fear of COVID-19 Scale (FCV-19S) [49] was used. The scale is valid in assessing fear of COVID-19. It contains seven items to which responses are given on a five-point rating scale (“strongly disagree,” “disagree,” “neither agree nor disagree,” “agree,” and “strongly agree”). Each item is assigned a score of 1–5, yielding a total score ranging from 7 to 35. The higher the score, the greater the fear of the coronavirus that may be experienced by the respondent.

The Polish adaptation of the scale proved a reliable and quick to use research tool with sound psychometric properties. The study confirmed that it could be successfully used in children and adolescents [49].

#### Children’s Depression Inventory 2 (CDI 2) [50, 51]

The Polish version of the CDI 2 self-report questionnaire [51] was employed in the study. This tool is commonly used to measure the severity of both emotional and functional problems in children and adolescents. The CDI 2 can aid in the identification of individuals at risk of depression, and treatment monitoring. In addition, this version offers four subscales for measuring negative mood, low self-esteem, ineffectiveness and interpersonal problems. The inventory includes 20 groups of answers. For each item, respondents are asked to endorse one of three statements that best describes their feelings and thoughts during the past two weeks. Responses are scored from 0 to 2. Converted T-scores are used to determine a trait profile of the respondent. Higher scores indicate higher levels of depressive symptoms. The Polish version of the inventory has high internal consistency and satisfactory reliability [51].

#### State-Trait Anxiety Inventory for Children (STAIC) [52, 53]

The STAIC test is used as a measure of state anxiety and trait anxiety in children. The first part of the questionnaire (C-1) was employed to evaluate transient situational anxiety ‘of now.’ It is composed of 20 statements. Each item has three possible response options: “yes,” “rather yes,” or “no”, which are scored from 1 to 3. The overall score of the summed-up items may be converted to T-score for interpretation. Higher scores correlate with greater anxiety. The internal consistency of the Polish version of the inventory is high [53].

#### Leyton Obsessional Inventory – Child Version (LOI-CV) [54–56]

The LOI-CV is designed as a tool for the identification of obsessive–compulsive symptoms and evaluation of their impacts on functioning. The Polish version of the inventory [55] was used in the study. The participants answered 20 questions by selecting “yes” or “no” responses, and, if the “yes” response was chosen, marked one of the four available options indicating the interference of the particular symptom with their everyday life. Each option scored 0 to 3. Both the number of the “yes” responses and the total interference score are relevant to the final result. The reliability of the Polish version is high for the entire inventory as well as individual items [55].

### Statistical analysis

Statistical analysis of the study results began with one-way analysis of variance (ANOVA) to verify whether the three groups differed from one another in FCV-19S PL, CDI 2, STAIC and LOI-CV scores. Then, a matrix of correlation coefficients between all variables was computed for each group. In the next step, complex two-factor interactions between groups and questions were verified. Finally, a self-developed multidimensional statistical model was applied. In addition, the Bartlett sphericity test was calculated, which was less than 0.001, and the Kaiser–Meyer–Olkin coefficient was 0.50. This proves the acceptability of the sample according to Kaiser [57]. All statistical tests were performed at the significance level, *α* = 0.05. Statistical analysis was conducted using IBM SPSS Statistics (Amos) and Statistica.

## Results

### Results of the basic statistical analysis

In the first step, differences in FCV-19S PL, CDI 2, STAIC and LOI-CV scores between the three groups were tested. The average scores and standard deviations are presented in Table 1.

**Table 1:** Descriptive statistics for FCV-19S PL, CDI 2, STAIC total score and LOI-CV by the three groups and overall

Variable	Statistic	Group			Overall
		Patients	Pupils	Children’s homes	
		*n* = 75; M = 20 (26.7%); F = 55 (73.3%)	*n* = 61; M = 27 (44.3%); F = 34 (55.7%)	*n* = 31; M = 13 (41.9%); F = 18 (58.1%)	*n* = 167; M = 60 (35.9%); F = 107 (64.1%)
FCV-19S	Mean	14.36	15.41	19.94	15.78
	SD	6.05	6.26	5.88	6.40
CDI 2	Mean	20.24	9.61	10.29	14.51
	SD	11.40	6.48	7.44	10.49
STAIC total score	Mean	37.77	27.66	28.94	32.44
	SD	11.22	7.26	8.64	10.59
LOI-CV	Mean	9.91	6.85	8.97	8.62
	SD	8.90	7.83	9.89	8.78

One-way analysis of variance (ANOVA) descriptive statistics are presented in Table 2.

**Table 2:** One-way ANOVA results for FCV-19S PL, CDI 2, STAIC total score and LOI-CV by the three groups

		**Degrees of Freedom**	**SS**	**MS**	**F**	***p***
FCV-19S PL	Intercept	1	39,858.437	39,858.437	1072.324	< 0.001
	Group	2	694.897	347.449	9.348	< 0.001
	Error	164	6095.905	37.170		
	Total	166	6790.802			
CDI 2	Intercept	1	25,989.728	25,989.728	308.849	< 0.001
	Group	2	4481.112	2240.556	26.626	< 0.001
	Error	164	13,800.624	84.150		
	Total	166	18,281.737			
STAIC total score	Intercept	1	143,658.823	143,658.823	1600.896	< 0.001
	Group	2	3910.302	1955.151	21.788	< 0.001
	Error	164	14,716.788	89.737		
	Total	166	18,627.090			
LOI-CV	Intercept	1	10,677.961	10,677.961	140.286	< 0.001
	Group	2	318.487	159.243	2.092	0.127
	Error	164	12,482.987	76.116		
	Total	166	12,801.473			

**Fig. 1:**
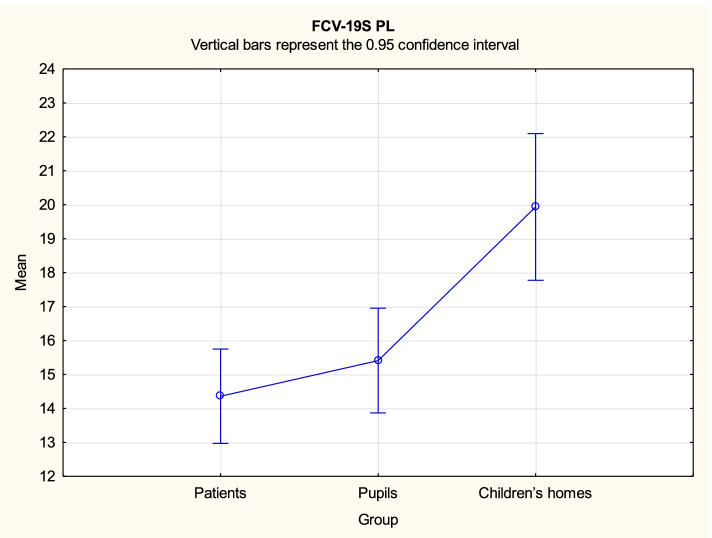
FCV-19S PL mean values with the related 95% confidence intervals by groups

**Fig. 2:**
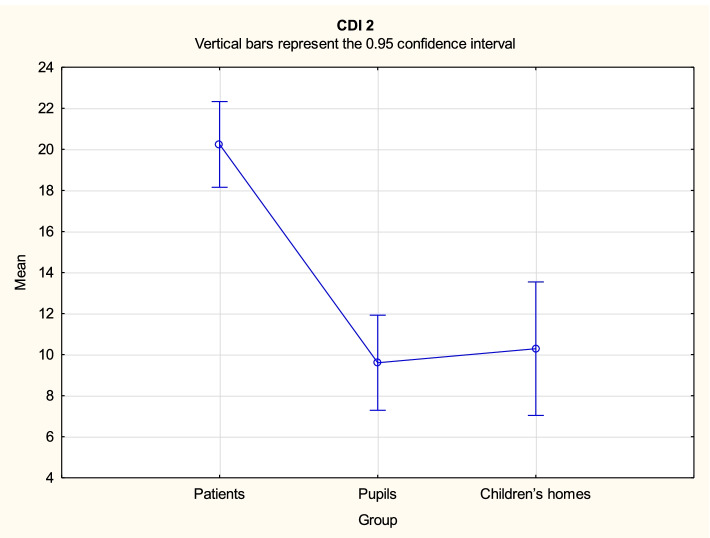
CDI 2 mean values with the related 95% confidence intervals by groups

**Fig. 3:**
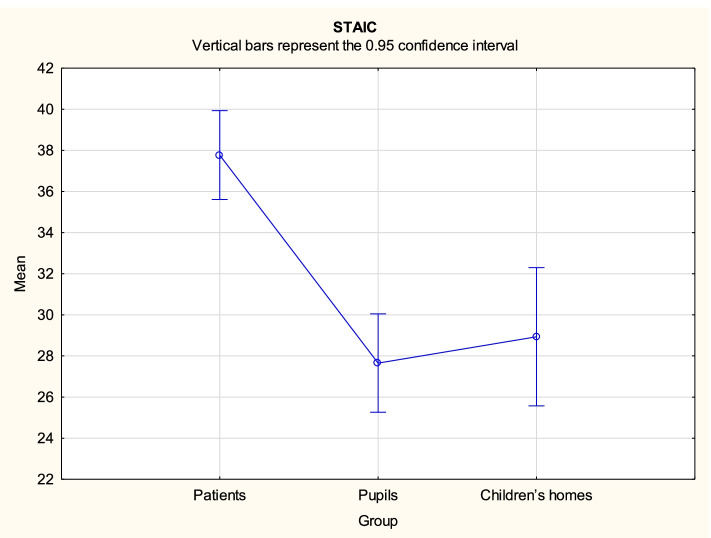
STAIC Total Score mean values with the related 95% confidence intervals by groups

**Fig. 4:**
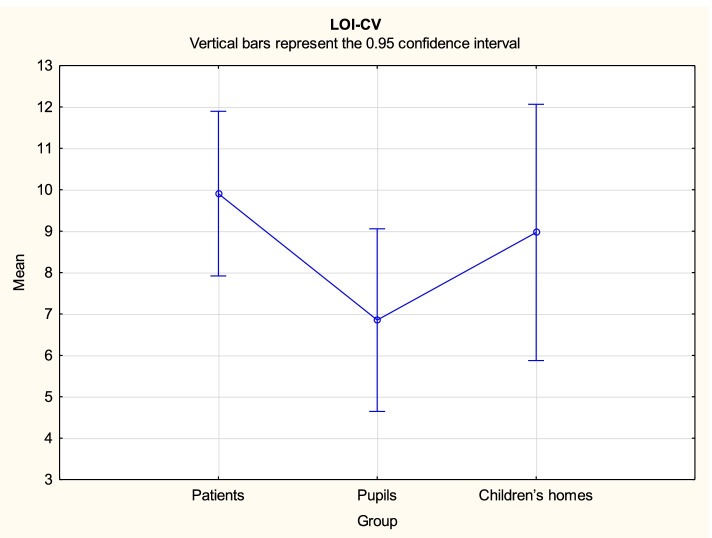
LOI-CV mean values with the related 95% confidence intervals by groups

Analysis demonstrated that there might be some statistically significant differences in variables FCV-19S PL, CDI2 and STAIC between the three groups. Duncan's post hoc tests were performed for all significant contrasts. The test results are provided in Tables 3, 4 and 5, while mean values with the related 95% confidence intervals are shown in Figs. 1, 2, 3 and 4.

**Table 3:** Duncan’s multiple range test results for differences in the variable FCV-19S PL between the three groups

Group		{1}	{2}	{3}
		14.36	15.41	19.94
1	Patients		0.397	< 0.001
2	Pupils	0.397		< 0.001
3	Children’s homes	< 0.001	< 0.001

**Table 4:** Duncan’s multiple range test results for differences in the variable CDI2 between the three groups

Group		{1}	{2}	{3}
		20.24	9.61	10.29
1	Patients		< 0.001	< 0.001
2	Pupils	< 0.001		0.714
3	Children’s homes	< 0.001	0.714

**Table 5:** Duncan’s multiple range test results for differences in the variable STAIC total score between the three groups

Group		{1}	{2}	{3}
		37.77	27.66	28.94
1	Patients		< 0.001	< 0.001
2	Pupils	< 0.001		0.506
3	Children’s homes	< 0.001	0.506

For the variable FCV-19S PL, the mean value was significantly higher for children from children’s homes (M = 19.94) compared to both pupils (M = 15.41) and patients (M = 14.36), while pupils and patients formed a statistically homogenous group in this respect.

For the variable CDI 2, the mean value was significantly higher for patients (M = 20.24) compared to both pupils (M = 9.61) and children from children’s homes (M = 10.29), while the latter two formed a statistically homogenous group in this respect.

For the variable STAIC Total Score, the mean value was significantly higher for patients (M = 37.77) compared to both pupils (M = 27.66) and children from children’s homes (M = 28.94), while the latter two formed a statistically homogenous group in this respect.

Statistics for the variable LOI-CV by groups are shown in Fig. 4. There are no statistically significant differences between mean values (patients: M = 9.91; pupils: M = 6.85; children from children’s homes: M = 8.97).

In the next step of analysis, the correlation matrix for questionnaire scores in each group and overall was computed. Pearson correlation coefficients, *r* are presented in Table 6.

**Table 6:** Pearson correlation coefficient matrix

		FCV-19S PL		CDI 2		STAIC Total Score		LOI-CV	
		*r*	*p*	*r*	*p*	*r*	*p*	*r*	*p*
Patients	FCV-19S PL	1		0.113	0.335	0.202	0.083	0.026	0.823
	CDI 2	0.113	0.335	1		0.814^**^	< 0.001	0.530^**^	< 0.001
	STAIC Total Score	0.202	0.083	0.814^**^	< 0.001	1		0.464^**^	< 0.001
	LOI-CV	0.026	0.823	0.530^**^	< 0.001	0.464^**^	< 0.001	1	
Pupils	FCV-19S PL	1		-0.040	0.760	-0.132	0.310	-0.037	0.774
	CDI 2	-0.040	0.760	1		0.542^**^	< 0.001	0.164	0.205
	STAIC Total Score	-0.132	0.310	0.542^**^	< 0.001	1		0.225	0.082
	LOI-CV	-0.037	0.774	0.164	0.205	0.225	0.082	1	
Children’s homes	FCV-19S PL	1		0.109	0.558	0.261	0.157	0.146	0.433
	CDI 2	0.109	0.558	1		0.751^**^	< 0.001	0.392^*^	0.029
	STAIC Total Score	0.261	0.157	0.751^**^	< 0.001	1		0.328	0.071
	LOI-CV	0.146	0.433	0.392^*^	0.029	0.328	0.071	1	
Overall	FCV-19S PL	1		-0.038	0.628	0.012	0.876	0.021	0.787
	CDI 2	-0.038	0.628	1		0.806^**^	< 0.001	0.417^**^	< 0.001
	STAIC Total Score	0.012	0.876	0.806^**^	< 0.001	1		0.390^**^	< 0.001
	LOI-CV	0.021	0.787	0.417^**^	< 0.001	0.390^**^	< 0.001	1	

*r* – Pearson correlation coefficient; *p* – significance of *r*;

^*^Correlation is significant at the *p* < 0.05 level

^**^Correlation is significant at the *p* < 0.01 level

The FCV-19S scores are not correlated with the CDI 2, STAIC and LOI-CV scores for each group and overall. The other three scales are positively correlated.

Further analysis aimed to determine which factors influenced the results in the three groups. To this end, all questions addressed to parents were reviewed. Their answers provided information on the children’s sociodemographic features (i.e. age, gender and size of the place of residence), and the epidemiological, economic and social status during the pandemic (parents’ professional status during the period, infections in the family, online learning, access to green areas, and psychological/psychiatric care provided to the child).

In order to investigate the correlations between children’s scores and answers to the aforementioned questions, descriptive statistics and the ratios of different responses by the group and overall were determined. This aimed to select the responses given in sufficient numbers to enable the two-way analysis of variance (ANOVA). The two-way ANOVA was performed in compliance with the requirements for applying statistical tests to samples of unequal size. In line with the established methodology, the data were analysed for an interaction effect first, and then for main effects.

The group and responses to individual items were found to produce no statistically significant differences in the four dependent variables. Consequently, the strongest differences may be interpreted for the study group only.

### Statistical model

Traditional statistical methods did not yield satisfactory results in this case. The two-way ANOVA failed to capture significant interactions. Therefore, a multidimensional statistical model was developed in an attempt to accurately explain the existing relationships.

The purpose of modelling was to investigate multidimensional relationships between sociodemographic features and the FCV-19S PL, CDI 2, STAIC and LOI-CV scores. The first major step was to identify a group of variables with the highest discriminant power over the entire set of features. In order to select the most powerful group (for building the most powerful model), various feature extraction methods were applied. Principal Component Analysis (PCA) is a process of using the kernel of a linear transformation to maximise the proportion of variance explained [58]. Kernel Principal Component Analysis (KPCA) is a non-linear extension of PCA [59]. Another method, Linear Discriminant Analysis (LDA), provides for partition into regions with linear functions [60]. Finally, the last two methods used for modelling were (i) Class-Centroid Principal Component Analysis (CCPCA), which involves the rotation of factors according to class centroids [61–63], and (ii) Gradient Principal Component Analysis (GPCA), in which stochastic gradient is applied to estimate the best rotation angle and the search step length [64].

The modelling sequence consists of the following major steps:

- To extract features using different methods and determine the discriminant power;
- To apply machine learning methods in order to develop the best prediction model;
- To build the optimum model for the best quality predictions.

The selection results for different feature extraction methods are presented in Table 7.

**Table 7:** Feature selection outcome for the five methods applied: proportion of variance explained and discriminant power of the extracted data set

Method	% of variance explained	Discriminant power of the set [0–1]
No extraction	61.12%	0.633
PCA	71.22%	0.748
KPCA	73.34%	0.772
GPCA	75.71%	0.811
CCPCA	77.28%	0.848
LDA	61.11%	0.647

**Fig. 5:**
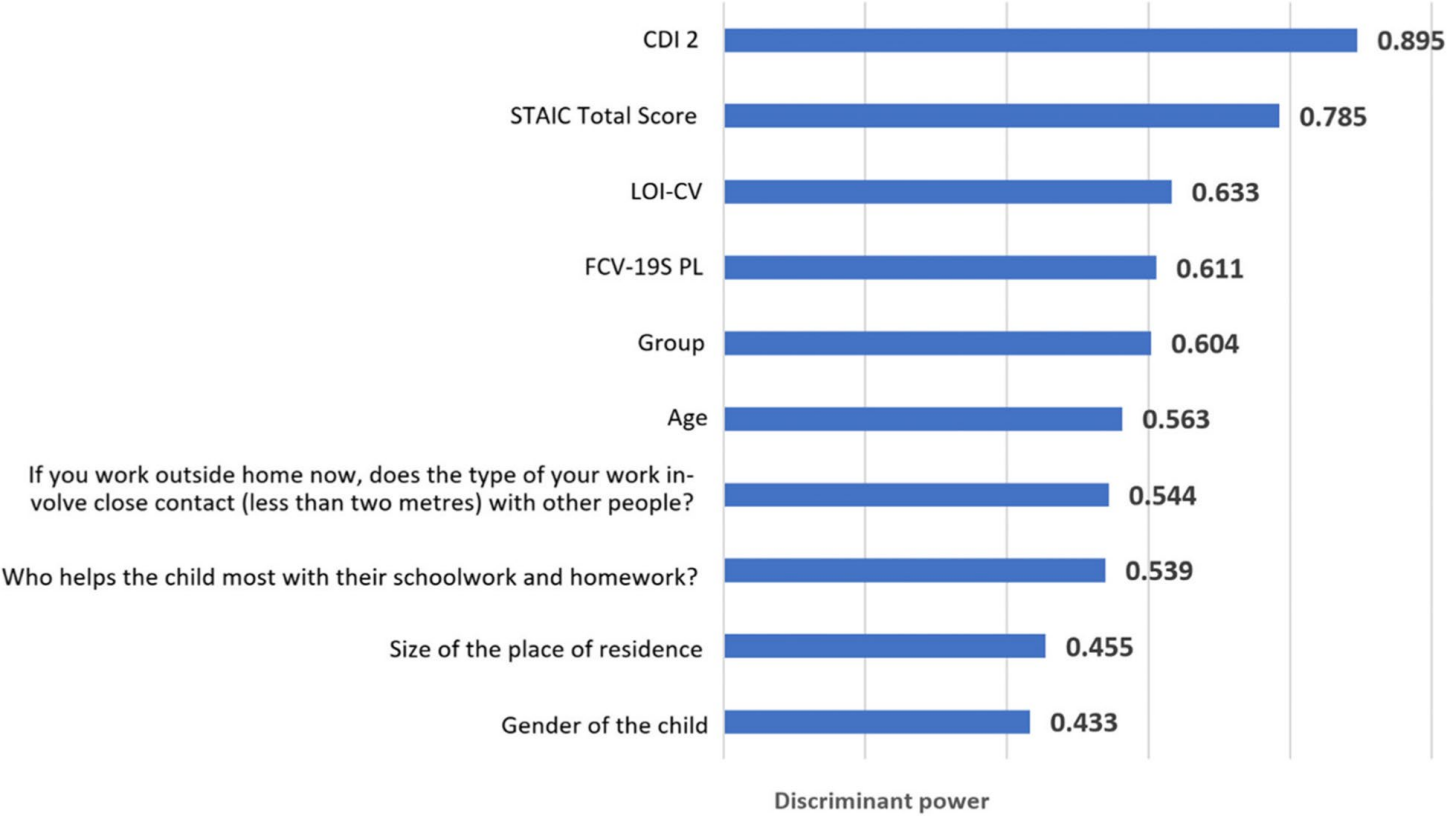
Discriminant power for the CCPCA extraction model

The highest proportion of variance explained (84.8%) was achieved using the CCPCA method. Consequently, this model was adopted for further tests. Notably, discriminant power was also the highest for this method. The features included in this set are presented in Fig. 5.

All features included in the model as statistically significant are shown in Fig. 5. These included: age, gender, size of the place of residence, child care, and work in close contact. Other features, such as infections in the family, parents/guardians’ employment status, access to green areas, learning model and psychological/psychiatric care, were found to be statistically insignificant.

**Fig. 6:**
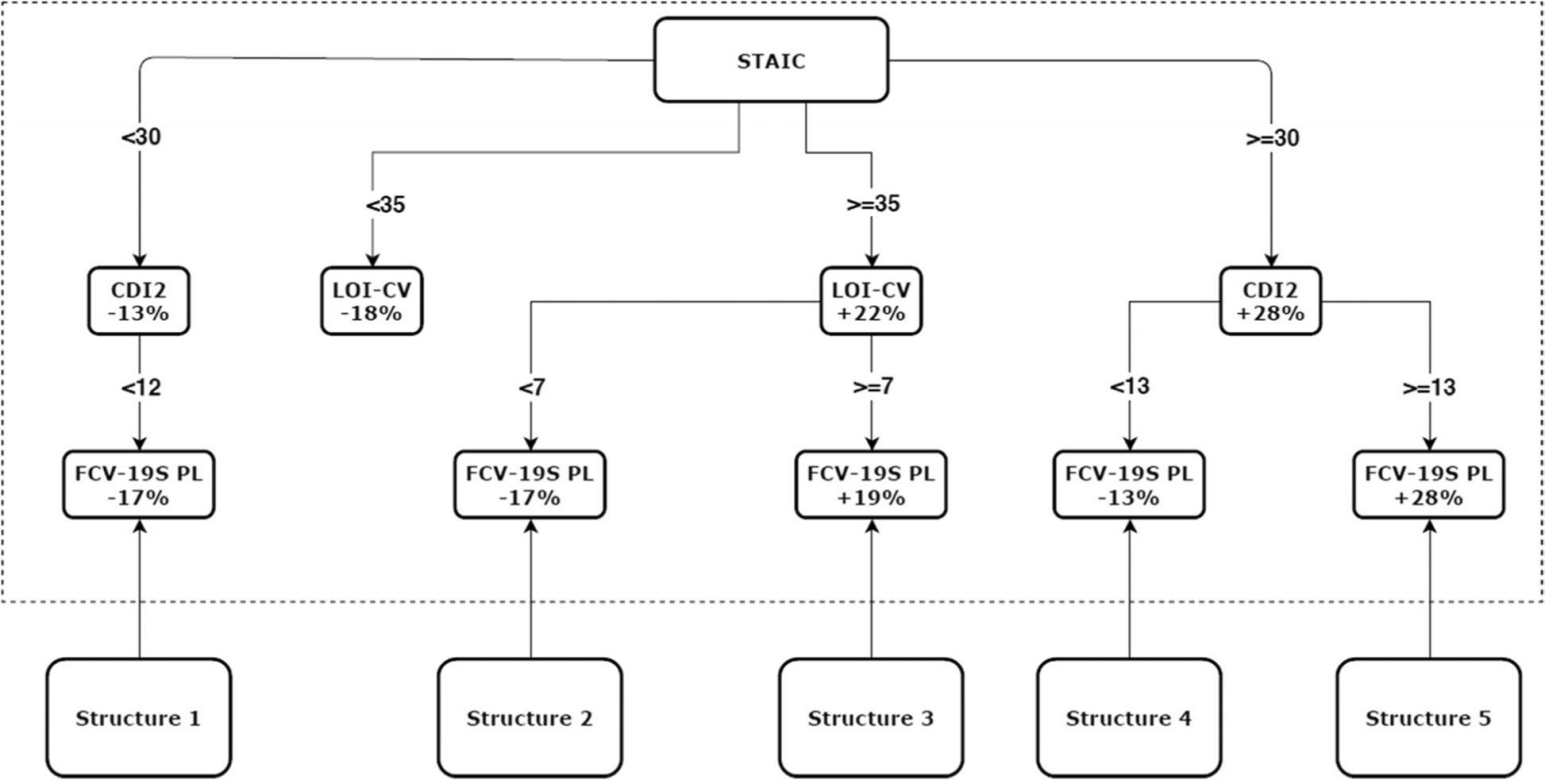
Classification And Regression Tree (CART)

The Classification And Regression Tree (CART) with the categorical variable structure are shown in Fig. 6 and Table 8.

**Table 8:** Posterior probabilities of the relevant structure categories in the CART

Variable	Response	Structure				
		1	2	3	4	5
If you work outside home now, does the type of your work involve close contact (less than two metres) with other people?	Yes	0.103	0.356	0.655	0.368	0.667
	No	0.897	0.644	0.345	0.632	0.333
Who helps the child most with their schoolwork and homework?	Parents	0.880	0.756	0.311	0.578	0.222
	Other persons	0.120	0.244	0.689	0.422	0.778
Year of birth of the child	2007 or earlier	0.504	0.478	0.417	0.578	0.323
	2008 or later	0.496	0.522	0.583	0.422	0.677
Gender of the child	Boy	0.543	0.450	0.610	0.450	0.633
	Girl	0.457	0.550	0.390	0.550	0.367
Place of residence	Village	0.456	0.510	0.402	0.412	0.324
	City	0.544	0.490	0.598	0.588	0.676
Group	Pupils	0.300	0.225	0.269	0.312	0.245
	Patients	0.376	0.388	0.387	0.455	0.411
	Children’s homes	0.324	0.387	0.344	0.233	0.344

In the model, if the STAIC score is greater than or equal to 30, the proportion of variation explained for the variable CDI 2 increases by 28%. Higher STAIC scores strongly support a 28% increase in CDI 2 scores.

Furthermore, for STAIC ≥ 30, if CDI 2 increased by 28% to ≥ 13, then FCV-19S also increased by 28%; else, for CDI 2 < 13, FCV-19S decreased by 13%. These tree branches are influenced by the factors included in the structure 5. There is a high probability that high values of the aforementioned variables are most often related to parents’ working in close contact with other people (*p* = 0.667) and child care provided by non-family members (*p* = 0.778). This situation is more frequent among children born in or after 2008 (*p* = 0.667), and more likely for boys (*p* = 0.633) and families living in cities (*p* = 0.676). Furthermore, this seems to occur most frequently in the patient group (*p* = 0.411).

For STAIC ≥ 30 and CDI 2 < 13, a decrease of 13% in FCV-19S was observed, when the parents/guardians’ type of work did not involve close contact (*p* = 0.632), when child care was provided by parents (*p* = 0.578), for children born before or in 2007 (*p* = 0.578), among girls (*p* = 0.550), and for people living in cities (*p* = 0.588). Such scores were also more likely in the patient group (*p* = 0.455).

Conversely, for STAIC < 30, the CDI 2 score decreased by 13%. For CDI 2 < 12, FCV-19S decreased by 17%. Low scores on these scales seemed to be definitely related with parents/guardians working without close contact (*p* = 0.897) and providing care at home (*p* = 0.880). This situation was slightly more frequent among children born before or in 2007 (*p* = 0.504), boys (*p* = 0.543), families living in cities (*p* = 0.544), and patients (*p* = 0.376).

Quite similar correlations were obtained for the STAIC and LOI-CV results. For STAIC ≥ 35, LOI-CV increased by 22%, then FCV-19S also increased by 19%. These results were observed, when parents’ working involved close contact with other people (*p* = 0.655) and child care was provided by non-family members (*p* = 0.689), for children born in or after 2008 (*p* = 0.583), more likely for boys (*p* = 0.610) and families living in cities (p = 0.598) and more often in the patient group (*p* = 0.387).

For STAIC ≥ 35 and LOI-CV < 7, FCV-19S was decreased by 17%. Such scores were more likely, when the parents/guardians’ type of work did not involve close contact (*p* = 0.644), when child care was provided by parents (*p* = 0.756), for children born before after 2008 (*p* = 0.522), among girls (*p* = 0.550), and for people living in villages (*p* = 0.510). These results were also more likely in the patient group (*p* = 0.388).

Notably, lower FCV-19S scores were always related to parents/guardians working without close contact and providing care at home, even if scores on other scales were high.

## Discussion

Our study aimed to investigate the impact of the SARS-CoV-2 pandemic on the psychological well-being of Polish children and adolescents diagnosed with psychiatric disorders.

Notably, children with a psychiatric diagnosis scored significantly higher on the CDI 2 and STAIC scales, while their FCV-19S and LOI-CV scores were not significantly different from those of the control group.

It is worth noting that in studies of other linguistic versions of the FCV-19S questionnaire among adults, scores on this scale were positively correlated with scores for other anxiety scales [48, 65, 66]. Interestingly, conversely to the results of previous studies on adult populations, our study did not find a correlation between FCV-19S and other fear related questionnaires. We consider this an important finding, which may suggest a different perception of the coronavirus among children. This seems highly plausible, considering magical thinking in children and developing causal reasoning ability in adolescents [67, 68]. For example, in a study of children aged 3–12 years in Spain, they often represented the coronavirus as an enemy that is being fought by the doctors (“our enemy the virus”) [18].

Our study confirmed the observed positive correlation between depression and anxiety symptoms during the COVID-19 pandemic and the severity of OCD symptoms [38, 41, 42]. However, classical statistical analysis did not show any significant differences in the severity of obsessive–compulsive symptoms between the group of patients, students and children from children’s homes. In the statistical model, high scores for anxiety and OCD symptoms were associated with children whose parents or guardians were exposed to close contact with other people at work and when the child was looked after by someone outside the family. This was not much more common in the group of patients, and another similar result was obtained by children from children’s homes. On the one hand, the lack of significant differences between the groups is not surprising because patients diagnosed with OCD are rarely treated in the hospital (our group of patients are people with a history of psychiatric hospitalization). On the other hand, taking into account the high comorbidity of psychiatric diagnoses with OCD [35, 36], one could expect significantly higher scores on the OCD diagnostic scales among patients compared to the control group. Previous studies have produced contradictory results as to the severity of obsessive–compulsive symptoms in the group of children and adolescents diagnosed with OCD [40, 46]. It is worth noting that each of these studies used different diagnostic tests. Patients also differed in access to follow-up their therapies during the pandemic. To further elucidate these discrepancies, additional longitudinal studies on larger groups would be needed.

Hitherto, only one study has in fact confirmed that a portion of visits to psychiatric services were directly related to the pandemic [47]. The prevalence of such visits was only 2%, yet the authors hypothesized that children and adolescents registered with a psychiatric diagnosis might be particularly vulnerable to symptom exacerbation during the pandemic. On a different note, it may be argued that children diagnosed with psychiatric disorders are typically provided with constant or regular psychiatric/psychological care and their carers tend to be more attentive and vigilant to their well-being. This may serve as a protective factor. Similar conclusions were provided by a study of the Danish population of children and adolescents diagnosed with OCD, in which a smaller increase in symptoms was observed in patients with constant and direct access to specialist care [41]. In our study, however, the responses regarding such care were not found to have a statistically significant impact.

According to the CCPCA model, the most potent protective factors, reducing the fear of the disease, were parental care and support, and lack of close contact at carers’ work. This finding is consistent with the data indicating a positive impact of harmonious family atmosphere, close relationships within the family and effective parent–child communication on the mental health of children during the COVID-19 outbreak [22, 24, 69]. Younger subjects frequently appreciated time spent with their parents [18]. The World Health Organization underscored in its guidelines and recommendations the role of parents in providing special care to children and ensuring mutual, open and honest communication during the pandemic [70].

In view of this evidence, it seems a logical conclusion that children from children’s homes, who lack individual parental care, were most prone to experience fear of the COVID-19. This group reached the highest scores on the questionnaire regarding fear of the disease. These children are also likely to have reduced access to information on the current epidemiological situation and to be more strongly affected by restrictions (carers’ distancing and a stricter sanitary regime due to a higher number of children and changing staff).

We also found that the FCV-19S score was influenced by the type of work performed by parents/guardians, specifically involving close contact (less than two metres) with other people. This is a particularly interesting outcome, as this factor is not directly related to children. The question was addressed to parents/guardians, so the responses are related to their subjective perception of the infection risk. Therefore, it seems likely that not only the type of virus information and data provided by adults but also their own level of fear may be influential. This conclusion would be consistent with the existing knowledge about the potent effects of parental modelling on children’s fear reactions [20, 71–73] and the role of threat information provided by parents in the anxiety development in children [9, 21, 74, 75].

Isolation is known to initially have a positive impact on the condition of some people with anxiety disorders, which include school phobias. Conversely, aggravation of anxiety-related symptoms in children with obsessive–compulsive disorder and symptom exacerbation in children with neurodevelopmental disorders (autism spectrum disorder – ASD or attention deficit hyperactivity disorder – ADHD) as a result of disturbance of daily routines were observed during the pandemic [47]. As the population of children and adolescents with psychiatric disorders is vastly heterogeneous, they might differ in their reactions to the epidemiological situation and its effects depending on their diagnosis. This group needs further research with diagnosis as a differentiating factor. Further studies in mental health centres could provide information on changes in visit frequency, number of hospitalisations, etc. It would be informative to observe changes in the severity of various symptoms over time; short questionnaires based on tools which are easy and fast to use, such as FCV-19S [48, 49], seem well-suited for this purpose. In the future, prospective matched-cohort studies would be needed to collect data and information on the impact of particular factors both during the exposure and post-exposure periods. It is crucial in view of potential long-lasting effects of the pandemic. Importantly, due to multiple risk factor exposure, the outbreak of COVID-19 may be the case of cumulative risk for children [69, 76]. The consequences of this situation may only become apparent at subsequent stages of development. Stress experienced during neurobiological development may be related to the perception of reality, stress coping [77] and depression in adulthood [78].

We would like to acknowledge some limitations to our study. Firstly, we obtained a low response rate in all groups, even though the study was targeted at a large number of children. The results of the Kaiser–Meyer–Olkin test and the Barlett's test show the acceptable sampling adequacy and the suitability of our data [57]. However, it is worth considering the possible reasons for the small number of respondents. At that time, schools were closed and learning was online. It was related to the fact that children had to spend many hours on the computer or smartphone. Therefore, in the case of a group of students and patients, the reason for the low number of questionnaires obtained could be the reluctance to spend extra time in front of monitors. The change in the learning mode may also have forced parents to monitor the progress of their children's work more intensively. As a result, they might not want to involve them in additional tasks. A similar situation could be associated with children from children’s homes. The pandemic has affected the way institutions operate. Employees may not have had enough time to become involved in our study and did not want to add additional responsibilities to the children. In addition, the subject matter of the study itself could discourage some from taking part in it due to the difficult topics of pandemic, stress, anxiety and depression as part of avoidance strategies. Another obstacle in conducting the study was the sanitary regime introduced in our country. This made direct contact with parents of students and guardians of children from children’s homes impossible. Despite the involvement of school principals and directors of children’s homes, we are not sure how many respondents actually received our questionnaires. There is also a risk that only children in whom parents suspect mental issues participated in the survey. This possibility should be given due consideration in further surveys. There is also a risk that the study mainly involved children whose parents or guardians suspected mental health problems. This possibility should be given due consideration in further research.

Secondly, according to the instructions included in the questionnaire, children should complete the questionnaires on their own without the supervision of their guardians. Unfortunately, the online form means that we have not been able to verify whether these conditions have been met. Children may have given inaccurate answers if they thought they would be read by the guardians or if the guardians were present when filling out the questionnaires. Therefore, it can be assumed that some of the survey results may be unreliable.

In addition, the groups were selected to match for age, but they emerged as heterogeneous in terms of gender (M/F: 35.9% vs. 64.1%).

The statistical model may be considered a strength of our study. It established relationships which were not clearly identified using basic methods.

## Conclusions

Our study adds to the understanding of the impact of the COVID-19 pandemic on the mental condition of children and adolescents, but there is a need for further research on this topic. Additional data on the factors that influence the perception of the virus, the disease and its effects by children, and on the extent to which various aspects of this phenomenon affect their mental well-being would facilitate fast and effective coping with the negative impact of the pandemic. In the future, it would also enable more accurate predictions of crucial problems arising in the case of disease or social isolation caused by outbreaks of communicable diseases or other issues. As a result, children and adolescents could be better protected against the negative effects thereof and the most vulnerable groups could be provided with special care.

## Data Availability

The datasets used during the current study are not publicly available due to participant confidentiality issues. They can be made available to the corresponding author on reasonable request.
